# Affliction’s Lonely Hour

**DOI:** 10.1111/criq.12717

**Published:** 2023-05-19

**Authors:** James Morland

**Affiliations:** ^1^ University of Sussex Brighton United Kingdom

It is two years since my diagnosis and I am still no closer to finding a reason why I have become so ill; I am an idiopathic medical anomaly. My diagnosis of chronic pancreatitis occurred in the middle of the first coronavirus lockdown in the United Kingdom. It was a solitary experience as I shuffled into distanced hospital waiting rooms, was fed into various machines that instructed me on when and how to breathe and sent back to my flat to await a phone call appointment to discuss their results. This solitary experience of illness was populated by many literary diagnostic voices. The words ‘no apparent cause’ were repeated across letters from my consultants to my GP surgery. As I was copied into an ongoing epistolary conversation about my health, it often felt like I was merely overhearing a conversation about me with no chance to interject. As if in response, the eighteenth‐century poets that I was researching gave their parallel diagnoses to my twenty‐first century consultants. While I had lost the energy to do most tasks, I spent the moments when my brain and body reconnected reading and taking notes on eighteenth‐century poems about health. If my consultants could not find the answer as to why I was ill, their eighteenth‐century counterparts pushed me to delve deeper inside my body and listen to my own rhythms of illness.

A 2016 study on chronic pancreatitis confirms that this could be a fruitful means of discovering why my pancreas has turned against itself. Presenting chronic pancreatitis as a ‘diagnostic dilemma’, it notes that it is ‘characterised by irreversible morphological change and typically causing pain and/or permanent loss of function’ and is ‘beset by destruction of healthy pancreatic tissue and the development of fibrous scar tissue’.^1^ The pancreas attempts to conceal itself by hiding behind the stomach and hampers research by being inaccessible and inflaming itself at the slightest touch. To understand the pancreas, it seems we need to reach beyond the medical and consult various sources. An article on the history of pancreatitis turns to the allegory of Plato’s cave in its conclusion, suggesting that those searching to understand the pancreas are the cave dwellers as ‘most of our knowledge of pancreatitis comes from the shadows cast by the disease’.^2^ We are always multiple steps behind the events that trigger the pancreas to destroy itself, attempting to understand its reasoning for doing so through the shadows cast by its inflammations. There is no universally accepted diagnostic standard for chronic pancreatitis; instead it is usually diagnosed with an array of radiological and endoscopic tools. But could poetic or emotional tools also be added to this list? An eighteenth‐century physician‐poet would argue that they were essential.

Mark Akenside’s 1745 *Odes on Several Subjects* are wide ranging, though there is a particular through line of the poet’s physical and emotional health across the collection. His personal experience of illness echoed my own pains and gave them a language: ‘How thick the shades of evening close! […] While rouz’d by grief these fiery pains / Tear the frail texture of my veins’.^3^ It might seem strange to find comfort in the works of an eighteenth‐century physician‐poet who had tried and failed multiple times to set up a successful practice and was known for his harshness towards patients, but his poetic bedside manner was one that spoke to me while in the depths of solitary episodes of chronic illness flareups.^4^

Akenside came to national fame for his poetry by the age of 22, though he also held a strong desire to continue a medical career path. He received a grant from his local dissenting church to study for the ministry, but within a year of arriving at the University of Edinburgh he had switched to study medicine. On returning to Newcastle by early 1742, he spent time working on his *The Pleasures of the Imagination*. Akenside sold the manuscript to the prominent bookseller Robert Dodsley for £120, and it appeared anonymously on 16 January 1744, swiftly becoming one of the most popular philosophical poems of the century. Notably, presumably with the money he had received from Dodsley, Akenside then went to Leiden University to further his medical studies. This medical education fed directly into his poetry, with Akenside seeing poetry as a therapeutic tool that could be used to ease the pains of mind and body.

After returning to England, he settled in Northampton and tried to establish a medical practice. After failing to entice enough patients away from the resident practitioner, he moved to Hampstead where his close friend Jeremiah Dyson owned a house. Dyson attempted to use his influence among the local residents to help Akenside establish a practice, but it once again failed and Akenside moved to Bloomsbury Square where Dyson provided him with a substantial allowance and a home.^5^ While being unsettled in his medical profession, Akenside was still prolific in his literary outputs with his *Odes on Several Subjects* published in March 1745.

Solitude appears throughout Akenside’s entire oeuvre, but it is the central focus of this collection of odes. The changing presentation of solitude follows the poetic meter of each ode, with a standardised iambic tetrameter or pentameter marking a solitary and introspective Horatian influence, with the speaker focusing on their relationship with nature and the potential perils of climate and ill health on one’s thoughts. The schemes of rhyming couplets in the Horatian odes act as a means for Akenside to explore various bodily and mental ailments. For Akenside, poetry is intensely therapeutic and is often the route towards health and pleasure. His rhymes attempt to replicate the physical effects of emotions and sickness and speak to how the rhythms of poetry itself could act as a cure.

‘Allusion to Horace’, the first ode of the 1745 collection, places the odes within a narrative of solitude and health. While confined in a London flat, this ode relocated me within a classical history of solitary gardens set in contrast to the crowded city:

Amid the garden’s fragrance laid,
Where yonder limes behold their shade
Along the glassy stream,
With Horace and his tuneful ease
I’ll rest from crouds, and care’s disease,
And summer’s piercing beam.^6^

Akenside presents a bee delighting in a natural withdrawal and the wealth of nature as his poetic muse, drawing an intimate relationship between the process of withdrawing from the crowds of the city and the production of both pleasure and health. The rhymed pairing of Horace’s ‘tuneful ease’ and ‘care’s disease’ is key to poetry and solitude’s curative quality, with Akenside setting Horace’s removal from crowds and his poetic rhythms as a cure from ‘care’s disease’. This is mirrored in the ending of the ode:

From all which nature fairest knows,
The vernal blooms, the summer rose,
She draws her blameless wealth;
And, when the generous task is done,
She consecrates a double boon,
To pleasure and to health.^7^

The ‘double boon’ of pleasure and health is a key motif across the volume of odes. The rhymed pairing of the ‘wealth’ of nature and the ‘health’ of the individual draws a neat parallel with the ‘ease’ of a Horatian ode providing respite from ‘care’s disease’ in the opening of the ode. The imagined solitary bower of the garden is a route to understanding how the ease of poetry and the wealth of nature combine to combat disease and encourage the health of both poet and reader.

This is placed specifically within Akenside’s mid‐century context as the bee refuses to fly near ruins and tombs in the sixth stanza:

Nor where the boding raven chaunts,
Nor near the owl’s unhallow’d haunts
Will she her cares imply;
But flies from ruins and from tombs,
From superstition’s horrid glooms,
To day‐light and to joy.^8^

Throughout the volume, there is a developing narrative transition from illness to health, focusing on the mind’s ability to will itself out of melancholy via thoughts of cheerfulness. Akenside sets his volume within the eighteenth‐century vogue for melancholy and solitary poetics, noting that his poetic muse steers more towards light and joy in its solitude rather than the ‘horrid glooms’ of the graveyard. The poem’s reference to the glooms of misty graveyards echoed my own day‐to‐day where the ‘double boon’ of pleasure and health seemed at a distance. I was sat within a brain fog that would not lift, suspended within my body’s ruins and the tomb of an organ that was atrophying. I was the ideal reader that Akenside was looking for. If I did not have the physical energy to pull myself off the sofa, perhaps his poetic muse could pull my mind towards daylight and joy.

Initially written in 1740 when Akenside was studying medicine at Edinburgh, the second ode, ‘On the Winter Solstice M.D.CC.XL.’ continues this transition from darkness to light. Based on Horace’s Ode 1.9, it adapts the original by incorporating specific solitary scenes and the griefs and melancholy of winter. Beginning as an ode to melancholy, it moves toward a celebration of mirth via the revelations that can be acquired in this wintery darkness. The ode opens in a similar manner to Horace, though Akenside is swift to introduce images of solitude and desolation in his British adaptation:

But lo, on this deserted coast
How faint the light! How thick the air!
Lo, arm’d with whirlwind, hail and frost,
Fierce winter desolated the year.^9^

Presenting the faint light of Britain's ‘deserted coast’, Akenside begins with an image of national isolation that serves as an apt setting for the solitary griefs of winter. Even Nature herself cannot escape the griefs of a British winter, which steadily infects the human population. After considering the religious revelations that winter can manifest, Akenside turns to the academic sitting lonely in his study within this desolate British winter. It was perhaps inevitable that I was to find my parallel figure in these solitary odes: a scholar of solitude following his archetype of *Il Penseroso* invoking melancholy in his lonely tower. Although Akenside suggests that this studious solitude is a key facet of my surviving illness’s ‘gloomy damps [that] oppress the soul’ by seeking refuge in my books^10^:

How pleasing wears the wintry night,
Spent with the old illustrious dead!
While, by the taper’s trembling light,
I seem those awful courts to tread
Where chiefs and legislators lie,
Whose triumphs move beyond my eye
With every laurel fresh‐display’d;
While charm’d I taste th’ Ionian song,
Or bend to Plato’s godlike tongue
Resounding thro’ the olive shade.^11^

While reading in solitude, Akenside’s speaker finds himself indulging in classical poetry and philosophy, allowing his imagination to take over and to construct an alternate scene to distract from the gloomy damps of winter. A solitary withdrawal into philosophy and poetry is followed by an imagined social interaction with these poets and philosophers of the past, just as my own reading of Akenside resulted in my solitary illness being spent convening ‘with the old illustrious dead’ to make sense of my ailing body. Reading poetry, then, has the power to populate our solitudes and distract us from our melancholy.

The third ode of the collection, ‘Against Suspicion’, suggested that my underlying pains could have been the bodily manifestations of my emotions. A personified Suspicion appears as a sorceress, ‘meditating plagues unseen’ on the body:

O Fly! ‘Tis dire Suspicion’s mien;
And, meditating plagues unseen,
The sorc’ress hither bends:
Behold her torch in gall imbrued:
Behold——her garments drop with blood
Of lovers and of friends.^12^

Suspicion’s torch is stained with gall, linking the emotion with its bodily ramifications. Perhaps this is why my gallbladder was being scanned by my medical team, using glowing dyes to trace years of misgivings. The poet becomes physician, noting the symptoms of the ‘secret venom’ from the unseen plagues and observing a ‘pale suffusion rise’ in their friend’s eyes:

Fly far! Already in your eyes
I see a pale suffusion rise;
And soon thro’ every vein,
Soon will her secret venom spread,
And all your heart and all your head
Imbibe the potent stain.

Then come the hours of shame and fear
Then hints of horror seize your ear;
While gleams of lost delight
Raise the deep discord of the brain,
As light’ning shines along the main
Thro’ whirlwinds and thro’ night.^13^

As my skin grew paler and my eyes glazed when I looked in the mirror, there was a diagnostic quality to Akenside’s lines that I had not encountered in medical language. Akenside relies on alliteration through these stanzas to enact the sequential poisoning effects of suspicion’s venom on the body. The ‘vein’ is paired with the ‘venom’ that infects it in the following line, while the ‘suffusion’ in the eyes is followed by its alliterated spread: ‘Soon will her secret venom spread’. Akenside continually relies on alliteration throughout the ode to poetically enact physical symptoms in the body. The ‘gleams of lost delight’ introduce another theme that reappears throughout the volume of odes: the detrimental effects of absence when not paired with a certain amount of cheerfulness. The gleams of lost delight serve to ‘Raise the deep discord of the brain’, with the alliteration between ‘delight’ and its ensuing ‘deep discord’ enacting the connection between the memory and the mental anguish such suspicion can cause. Similarly, the repetition of ‘all your heart and all your head’ connects with the initial symptom ‘Already in your eyes’, with Akenside using assonance and alliteration to document the overwhelming and interweaving quality of an illness as it overtakes your body. These vowels gave a language to the feeling of the unseen potent strain of lethargy that was slowly seeping into every joint of my body. In encouraging me to withdraw into my body's own rhythms, Akenside was helping me to build a vocabulary of what it felt like to have an illness that seemed to slowly overwhelm my body.

Akenside’s fifth ode, the ‘Hymn to Cheerfulness, The Author Sick’ opens with a familiar scene within the thick ‘shades of evening’ of midwinter.^14^ I saw myself within this poem most clearly, with its depiction of the speaker in a solitary sickroom, his mind following the melancholy paths he has warned his readers against in his previous odes. Much like in Ode II, nature cannot soothe the mind’s melancholy, and a remedy must come from elsewhere:

Is there in nature no kind pow’r
To sooth affliction’s lonely hour?
To blunt the edge of dire disease,
And teach these wintry shades to please?
Come, Cheerfulness, triumphant fair,
Shine thro’ the painful cloud of care;
O sweet of language, mild of mien,
O virtue’s friends and pleasure’s queen!^15^

At a time where there was no immediate remedy to my illness, it was easy to become enraptured by this call to the power of poetry as a way of populating my own affliction’s lonely hour. Cheerfulness is linked to the therapeutic quality of poetry by promoting a healthy mind and body through poetic rhythm. Akenside furthers this connection of the offices of medicine and poetry through his description of cheerfulness:

Thou, Cheerfulness, by heaven design’d
To rule the pulse, that moves the mind,
Whatever fretful passion springs,
Whatever chance or nature brings
To strain the tuneful prize within,
And disarrange the sweet machine,
Thou, Goddess, with a master‐hand
Dost each attempter’s key command,
Repine the soft and swell the strong,
Till all is concord, all is song.^16^

Amid his definitions of cheerfulness and the related benefits of keeping oneself between extremes, Akenside is keen to show how it can therapeutically benefit those who find themselves in an illness‐induced solitude. Akenside consistently relies on the dual meaning of ‘composing’ throughout the ode, used in the sense of both poetic composition and the calming and quieting of the mind. Just as in poetry, the body’s rhythms can be composed so that ‘all is [a] song’ of health. His sickroom is a place where thoughts move inwards and towards one’s own mortality, where ‘yon deep death‐bell’s groaning sound / Renew my mind’s oppressive gloom, / Till starting horror shakes the room’.^17^ Here, Akenside calls on a long tradition of the solitary sickroom being a space where the mind dwells on the body and its corporeality, notably John Donne's call from his sickbed that ‘never send to know for whom the bell tolls; it tolls for thee’.^18^ Within the sickroom, at the mercy of ‘affliction’s lonely hour’, Akenside calls to Cheerfulness to ‘blunt the edge of dire disease’.^19^ Alliteration and poetic rhythm are again used to document the mental pains of illness and the ensuing curative effects of cheerfulness on the solitary mind and body. In the ‘dull, dejected scene’ the speaker hears deep death‐bells and feels the edges of ‘dire disease’, with alliteration mimicking the feelings of repetition and never‐endingness that come with chronic illness. The apostrophe ‘Come, Cheerfulness’ acts as an alliterative intervention and poetic counterpoint to the ‘cloud of care’, marking its therapeutic countering of this melancholic downturn.

Soon after Cheerfulness appears in the ode, four apostrophising lines mark its stable and steady rhythmic intervention into the affliction’s lonely hour:

O sweet of language, mild of mien,
O Virtue’s friend and pleasure’s queen!
Asswage the flames that burn my breast,
Attune my jarring thoughts to rest.^20^

Cheerfulness begins to take hold through the same alliteration that had exemplified the dire quality of illness, wrestling with disease and offering ‘gracious gifts’ to assuage ‘the flames that burn my breast’.^21^ The ‘attune’ is key to understanding Akenside’s attempts at combining his poetic and medical expertise. Francis Bacon, in his *Of the Advancement of Learning*, suggests that the harmony of poetry aligns directly with the ‘office of medicine’: ‘the Poets did well to conjoyne Musicke and Medicine in Apollo, because the Office of Medicine, is but to tune this curious Harpe of mans bodie, and to reduce it to Harmonie’.^22^ As the ‘sweet of language’, poetry has the power to give language to our bodies’ distempers but also to actively ‘attune’ our ‘jarring thoughts to rest’ and bring the body as a whole to a rhythmic harmony so that ‘all is song’.

Through the course of the ode collection, Akenside urged me to find this cheerfulness that would wrestle the alliterative repetitiveness of my illness. Akenside ends the poem by calling to cheerfulness to share its place with the poetic muse in our minds and to ‘sooth to peace corroding care’, a reference back to the similarly alliterated ‘cloud of care’ at the beginning of the ode.^23^ At this point, Akenside anticipates my developing cynicism at the concept of finding a pure cheerfulness in the face of a sickness that overwhelms every fibre of my being. As Stuart M. Tave has noted, ‘cheerfulness’ was a key word of the eighteenth century, with Joseph Addison and Richard Steele often vouching for its beneficial nature.^24^ Akenside’s description of cheerfulness is perhaps inspired by Joseph Addison’s formulations of cheerfulness in *The Spectator*. Akenside’s reference to the ‘sweet machine’ of the human body echoes the practical benefits of cheerfulness as written by Addison in *The Spectator No. 387*: ‘Cheerfulness is, in the first place, the best Promoter of Health. Repinings, and secret Murmurs of Heart, give imperceptible Strokes to these delicate Fibres of which the Vital Parts are composed, and wear out the Machine insensibly’.^25^ As the child of Love and Health, Akenside’s cheerfulness is similarly the best promoter of health as it has the power to turn the disarranged tunes of the human body back into a steady song. Akenside’s ode, with its shift from the melancholic throes of illness to the controlled song of health also seemingly pulls from Addison’s writings on cheerfulness in *The Spectator No*. *381*: ‘I have always preferred Cheerfulness to Mirth: Cheerfulness does not give the mind such exquisite gladness as mirth, but it is not liable to a succeeding melancholy depression; it filled the mind with a steady and perpetual serenity’.^26^ Addison’s piece begins with the Epicurean inspired opening lines of Horace’s Ode 2.3: ‘An equal mind, when storms overcloud, / Maintain, nor ‘neath a brighter sky / Let pleasure make your heart too proud, / O Dellius, Dellius! Sure to die’.^27^ It is best to remain with a steady and equal mind in order to enjoy life before inevitable death, Epicurus argued. Horace’s opening lines reworked the Epicurean maxim that ‘man is unhappy either because of fear or because of unlimited and groundless desire; and by reigning these in he can produce for himself the reasoning [which leads to] blessedness’, calling for a levelheadedness when met with extremes.^28^ I had spent years researching eighteenth‐century reinterpretations of Epicureanism while being plagued by illnesses that threw me off this equilibrium and into the extremes. After years of pain‐filled pancreatic attacks, I was left with a body that could no longer function because of this imbalance. Like the solitary scholar in Akenside’s second ode, I turned to poets and philosophers to populate my imagination and to convene with the ‘illustrious dead’ to find a way to regain an equilibrium in a body that was off kilter. With his conception of cheerfulness, Akenside stepped in to provide his diagnosis and cure. His controlled poetic rhythm and consistent use of alliteration was a route towards a steadier serenity.

The ending of the ode serves as a marker for the healing qualities of a poetic cheerfulness, but also a note of the acceptable levels of woe that one can allow in the face of absence:

I court the muse’s healing spell
For griefs that still with absence dwell,
Do thou conduct my fancy’s dreams
To such indulgent, tender themes
As just the struggling breast may cheer,
And just suspend the starting tear,
Yet leave the charming sense of woe,
Which none but friends and lovers know.^29^

Cheerfulness is the mediator between extremes of pleasure and pain. Poetry can be a ‘healing spell’ for the solitary pains of absence, with cheerfulness able to conduct the imagination to some ‘tender themes’ and to suspend tears. But what is left is a ‘charming sense of woe’ that those who have experienced friendship and love know. In conversations about health, there is also a sense of woe which none but the chronically ill know.

At this point in the collection, Akenside suggests that I should have developed some confidence in my own solitude. Unlike Ode II, this ode does not end with solitude being interrupted by the entrance of a friend but instead with a celebration of thoughts of friendship that can exist within solitude. The poet has advanced in his ability to cope with solitude through a stronger reliance on imagination. The melancholic throes at the beginning of the ode are balanced by the speaker’s inner resources courtesy of the successful invocation of alliterative cheerfulness, rather than through the intervention of a friend.^30^ The pleasures of the sociability of being a friend or a lover are balanced by their absence to produce a form of tempered melancholy that populates solitude with a knowledge of the complementary benefits of cheerfulness and melancholy. All well and good, but can the illnesses that punctuated this collection also be balanced purely by the thoughts of a body that once was?

To quote a letter sent by Akenside to Jeremiah Dyson while suffering melancholy in Leiden, he sought to advance a concept of a desirable ‘good health’ that is encapsulated by ‘that inexplicable *feeling* that *we are happy’*.^31^ Poetry is how Akenside could define and depict that feeling. The final odes of the collection go some way to explaining the boundaries and limits of poetry’s capabilities in that endeavour. Initially, the speaker concentrates on the healing benefits of sleep as a ‘silent pow’r’ and Morpheus as the ‘God of kind shadows and of healing dews’, before apostrophising to Sleep: ‘O let not me thus watch alone! / O hear my solitary moan!’.^32^ While the rest of the ode reflects on the varied experiences of dreaming, the final stanza of this ode returns to Akenside’s overarching aim to combine the healing qualities of poetry and medicine. In his solitary sleepless state, the speaker appeals to both poetry and health in his final apostrophe to Morpheus, god of sleep and dreams:

But, Morpheus, on thy dewy wing
Such fair auspicious visions bring,
As sooth’d great Milton’s injured age,
When in prophetic dreams he saw
The tribes unborn with pious awe
Imbibe each virtue from his heavenly page:
Or such as Mead’s benignant fancy knows,
When health’s kind treasures, by his art explore,
Have saved the infant from an orphan’s woes,
Or to the trembling sire his age’s hope restored.^33^

The reference to Richard Mead, the physician to whom Akenside dedicated his M.D. thesis, and John Milton near the end of the odes encapsulates the equal footing that both poetry and medicine have held throughout the volume.^34^ As Sleep provides its ‘healing dews’, it brings ‘fair auspicious visions’ of both poetry and medicine to the solitary poet, who seeks (and has sought through the volume) to promote a sense of physical, mental, and moral well‐being through his own image. I was perhaps too keen to find diagnoses and cures in poetry. It, of course, cannot heal an organ that is withering within my body. Akenside reminds me that ‘health’s kind treasures’ are more immediate in their healing of woes, something mainly medicine and medication can take care of. But the reference to Milton’s ‘prophetic dreams’ of the ‘tribes unborn’ suggests that poetry’s soothing qualities are instead linked to a sense of futurity. The alliterative repetitions of illness are also found in the cyclical comings of spring. The composition of poetry and the body and mind are closely intertwined for Akenside. A well‐composed poem was a means to document the ill body and to compose and calm the mind. Its rhythms could map a pain‐filled body in ways that medical language could not. Its compositional elements could be used to instruct the reader on how to help themselves out of their solitary woes through the power of imagination and fancy. This poetic imagination might not heal the body, but it produces visions of a future beyond an illness.

My medical diagnosis left me with an incomplete sense of my body and its failings, placing me within affliction’s lonely hour. Stepping away from the purely medical and considering my body through the language of poetry allowed me to exist within a space where I could engage fully with my body’s communications, whether through a brain fog or a stabbing pain. While Akenside might not have been able to diagnose me, his lines encouraged me to indulge my body’s calls for pauses, to sit in its fog and map out its interiors. Instead of interpreting MRI scans, we worked together to map the rhythms of my body. The rhythms and couplets of these poems helped situate myself within my solitude and within my body to develop a language that made sense of my foggy future existence. Alliterations became my body language, marking my body's decline from the tightknit couplets of health to the irregular poetic meter of illness in a way that served to ‘blunt the edge of dire disease’.

## Notes

1 Sinead Duggan, et. al, ‘Chronic pancreatitis: a diagnostic dilemma’, *World Journal of Gastroenterology*, 22:7 (2016), p. 2305.

2 D. A. O’Reilly & A. N. Kingsnorth, ‘A brief history of pancreatitis’, *Journal of the Royal Society of Medicine*, 94 (2001), pp. 130‐2.

3 Mark Akenside, ‘Ode V. Hymn to Cheerfulness, *The Author Sick*’ in *Odes on several subjects* (London: Printed for R. Dodsley. And sold by M. Cooper, 1745), lines 5‐8.

4
*The Poetical Works of Mark Akenside, Edited with a Life by Rev. Alexacnder Dyce* (Boston: Little, Brown and Company, 1863), p. 70.

5 Robin Dix, *The Literary Career of Mark Akenside* (Madison: Fairleigh Dickinson Univesrity Press, 2006), p. 22.

6 Akenside, ‘Ode I. Allusion to Horace’, 1‐6.

7 Ibid., 43‐48.

8 Ibid., 31‐36.

9 Akenside, ‘Ode II. On the Winter‐Solstice, MDCCXL’, 11‐24.

10 Ibid., 20.

11 Ibid., 61‐70.

12 Akenside, ‘Ode III. Against Suspicion’, 1‐6

13 Ibid., 7‐18.

14 Akenside, ‘Ode V. Hymn to Chearfulness. *The Author Sick*’, 1.

15 Ibid., 13‐20.

16 Ibid., 47‐56.

17 Ibid., 10‐12.

^18^
John Donne, ‘Meditation XVII’ in *Devotions upon Emergent Occasions* (Cambridge: Cambridge University Press, 1923), p. 98.

19 Ibid., 15.

20 Ibid., 19‐22.

21 Ibid., 21‐3.

22 Francis Bacon, *The Twoo Bookes of Francis Bacon: Of the proficience and aduancement of Learning, diuine and humane* (London: Printed for Henrie Tomes, and are to be sold at his shop at Graies Inne Gate in Holborne, 1,605), p. 38‐9.

23 ‘Ode V’, 142.

24 Stuart M. Tave, *The Amiable Humorist* (Chicago: Chicago University Press, 1960), p. 9.

25 Joseph Addison, *Spectator No. 387*, quoted in Robin Dix, *The Literary Career of Mark Akenside* (Madison: Fairleigh Dickinson University Press, 2006), p. 165.

26 Addison, *Spectator No. 381*, quoted in Tave, *The Amiable Humorist*, p. 9.

27 Ibid.

28 Porphyry, *To Marcella* 29, in *The Epicurus Reader*, trans. and eds. Brad Inwood and Lloyd P. Gerson (Indianapolis: Hackett, 1994), p. 103; C. W. MacLeod, ‘Ethics and Poetry in Horace’s Odes (1.20; 2.3), in *Greece & Rome*, 26:1 (April 1977), pp. 25‐6.

29 ‘Ode V’, 153‐60.

30 Dix, *Literary Career*, p. 167.

31 Mark Akenside to Jeremiah Dyson, Leyden, April 21st, 1744, in *Literary Career*, p. 300.

32 Akenside, ‘Ode IX. To Sleep’, 1, 8, 19‐20.

33 Ibid., 49‐58.

34 Dix, *Literary Career*, p. 171‐2.

